# Assess PET/MR in diagnosis of disease in comparison with PET/CT

**DOI:** 10.1186/2197-7364-2-S1-A69

**Published:** 2015-05-18

**Authors:** Jianhua Yan, Jason Chu-Chern Lim, Hoi Yin loi, John Totoman, Arvind Kumar Sinha, Swee Titan Quek, David Townsend

**Affiliations:** A*STAR-NUS Clinical Imaging Research Centre, Singapore; Department of Diagnostic Imaging, National University Hospital, Singapore

The aim of this study is to assess the performance of 18F-FDG whole body PET/MRI in comparison with PET/CT based on SUV. Anatomical location of lesion with Dixon MRI and additional value of advanced MRI technology such as diffusion weighted MR imaging in diagnosis of malignant disease will also be investigated.

